# Mechanisms of haplotype divergence at the *RGA08 *nucleotide-binding leucine-rich repeat gene locus in wild banana *(Musa balbisiana)*

**DOI:** 10.1186/1471-2229-10-149

**Published:** 2010-07-16

**Authors:** Franc-Christophe Baurens, Stéphanie Bocs, Mathieu Rouard, Takashi Matsumoto, Robert NG Miller, Marguerite Rodier-Goud, Didier MBéguié-A-MBéguié, Nabila Yahiaoui

**Affiliations:** 1 CIRAD, UMR DAP, TA A-96/03, Avenue Agropolis, F-34398 Montpellier Cedex 5, France; 2 Bioversity International, Parc Scientifique Agropolis II, F-34397 Montpellier Cedex 5, France; 3 Rice Genome Research Program (RGP), National Institute of Agrobiological Sciences (NIAS)/Institute of the Society for Techno-innovation of Agriculture, Forestry and Fisheries, Tsukuba, Ibaraki 305-8602, Japan; 4 Postgraduate program in Genomic Science and Biotechnology, Universidade Católica de Brasília, SGAN 916, Módulo B, CEP 70.790-160, Brasília, DF, Brazil; 5 Universidade de Brasília, Campus Universitário Darcy Ribeiro, Instituto de Ciências Biológicas, Departamento de Biologia Celular, Asa Norte, Brasília, Brazil; 6 CIRAD, UMR QUALITROP, F-97130 Capesterre-Belle-Eau, Guadeloupe, France

## Abstract

**Background:**

Comparative sequence analysis of complex loci such as resistance gene analog clusters allows estimating the degree of sequence conservation and mechanisms of divergence at the intraspecies level. In banana (*Musa sp*.), two diploid wild species *Musa acuminata *(A genome) and *Musa balbisiana *(B genome) contribute to the polyploid genome of many cultivars. The *M. balbisiana *species is associated with vigour and tolerance to pests and disease and little is known on the genome structure and haplotype diversity within this species. Here, we compare two genomic sequences of 253 and 223 kb corresponding to two haplotypes of the *RGA08 *resistance gene analog locus in *M. balbisiana *"Pisang Klutuk Wulung" (PKW).

**Results:**

Sequence comparison revealed two regions of contrasting features. The first is a highly colinear gene-rich region where the two haplotypes diverge only by single nucleotide polymorphisms and two repetitive element insertions. The second corresponds to a large cluster of *RGA08 *genes, with 13 and 18 predicted RGA genes and pseudogenes spread over 131 and 152 kb respectively on each haplotype. The *RGA08 *cluster is enriched in repetitive element insertions, in duplicated non-coding intergenic sequences including low complexity regions and shows structural variations between haplotypes. Although some allelic relationships are retained, a large diversity of *RGA08 *genes occurs in this single *M. balbisiana *genotype, with several *RGA08 *paralogs specific to each haplotype. The *RGA08 *gene family has evolved by mechanisms of unequal recombination, intragenic sequence exchange and diversifying selection. An unequal recombination event taking place between duplicated non-coding intergenic sequences resulted in a different *RGA08 *gene content between haplotypes pointing out the role of such duplicated regions in the evolution of RGA clusters. Based on the synonymous substitution rate in coding sequences, we estimated a 1 million year divergence time for these *M. balbisiana *haplotypes.

**Conclusions:**

A large *RGA08 *gene cluster identified in wild banana corresponds to a highly variable genomic region between haplotypes surrounded by conserved flanking regions. High level of sequence identity (70 to 99%) of the genic and intergenic regions suggests a recent and rapid evolution of this cluster in *M. balbisiana*.

## Background

Comparative genomics studies at the interspecies and intraspecies levels have revealed the dynamics of genome evolution and the plasticity of plant genomes. Within monocotyledons, the grass species of the order Poales have been extensively studied for their genome structure and evolution [1], but little is known about monocotyledon plants outside this group. Musa species are giant herbs from the order Zingiberales of the monocotyledon group and comprise two important tropical crops, banana and plantain. Two wild diploid Asian *Musa *species, *M. acuminata *Colla (A genome, 2n = 2x = 22) and *M. balbisiana *Colla (B genome, 2n = 2x = 22) are at the origin of most cultivated bananas. Intraspecific hybridizations within *M. acuminata *and interspecific hybridizations with *M. balbisiana *have resulted in various combinations of the A and B genomes in different cultivars. A majority of these cultivars are triploids with a genome constitution of AAA (mostly dessert banana), AAB (including plantains) and ABB [2]. Limited genetic variation in the most economically important banana cultivars, which are clonally propagated sterile polyploids, has resulted in a crop lacking resistance to some major fungal, bacterial and viral pathogens and to several pests.

Wild *M. balbisiana *is native to South Asia with a geographical distribution ranging from India to south China [3,4]. It grows in forest clearings and produces non-pulpy, non-edible bananas. Plants of this species are immune to several diseases and pests such as Fusarium wilt and Black leaf streak disease and represent excellent sources of natural resistance [5,6]. The B genome is used in breeding programs for its traits of vigour, of cold resistance [7] and could also be interesting for tolerance to pests and pathogens. Recent studies involving *M. balbisiana *plants prospected in China have shown that significant genetic diversity exists in this species [8]. A better knowledge of the genome structure and diversity of *M. balbisiana *should help exploiting this resource to increase the genetic basis of cultivated bananas.

Genomic resources have been recently developed to study the genome structure of *Musa *species. The size of the haploid *Musa *genome is estimated as varying between 560 to 600 Mb [9,10]. BAC (bacterial artificial chromosome) libraries for *M. acuminata *[11,12] and *M. balbisiana *[13] are available and analysis of BAC end sequences from *M. acuminata *"Calcutta 4" determined a gene density of one per 6.4 kb, similar to that of rice [14]. Further insight on the *Musa *genome structure was recently provided by the sequencing and analysis of 1.8 Mb from 17 BAC clones [15]. The 443 predicted genes revealed that Zingiberales genes share GC content and distribution characteristics with Eudicot and Poaceae genomes. Comparison with rice identified traces of microsynteny that were retained in several regions since the divergence of the Poales and Zingiberales at least 117 MYA. Comparisons of two genomic regions representing 140 kb from *M. acuminata *and *M. balbisiana *revealed a highly conserved genome structure, and indicated that these genomes diverged circa 4.6 Mya [15]. However, no data is yet available on *Musa *intraspecies sequence diversity and divergence.

Resistance (*R*) genes encode proteins that indirectly or directly detect specific pathogen avirulence genes products thus triggering a resistance response [16]. The largest class of *R *genes encodes proteins with a nucleotide binding domain and leucine-rich repeats (NB-LRR proteins) associated at the N-terminal to a Toll/Interleukin-1 Receptor (TIR) homologous region or to a coiled-coil (CC) motif. NB-LRR encoding genes have been widely studied in plant genomes [17-20]. Most of them are organized in arrays of closely related genes resulting from tandem duplication events. NB-LRR gene clusters have been identified in several plant species, e.g. in lettuce [21], potato [22], rice [23,24], wheat [25] and common bean [26,27]. They can span from less than a hundred Kb up to several megabases, as in the *RGC2 *locus in lettuce [21]. Within clusters, sequence exchange through unequal crossing over or gene conversion leads to the formation of new chimeric genes. In addition, point mutations and diversifying selection result in the creation and maintenance of novel *R *gene variants. Studies on the *Rpp5 *cluster in Arabidopsis, the *R1 *locus in potato and the *Rpg1-b *locus in soybean have shown reduced colinearity and differences in NB-LRR gene numbers between haplotypes, likely due to unequal crossing over between paralogs [22,28,29].

In banana species, around two hundred and fifty resistance gene analog (RGA) partial sequences have been amplified using primers from conserved motifs of NB-LRR genes [30-33]. Recent work identified the partial MaRGA08 sequence from the wild diploid *M. acuminata *Calcutta 4 [33]. Used as a hybridization probe, this sequence revealed the presence of a multigenic family across three *Musa *genomes, including the wild diploid *M. balbisiana*. Here, we have sequenced, annotated and compared *M. balbisiana *"Pisang Klutuk Wulung" (PKW) BAC clones showing high densities of this NB-LRR RGA. These sequences corresponded to two haplotypes of *M. balbisiana *which allowed us to study intraspecific variation of this wild *Musa *species at the molecular level. New Musa repetitive elements have been identified, and the structure and evolution mechanism of a large cluster of NB-LRR genes identified in these sequences is revealed.

## Results

### Identification of two large clusters of the *RGA08 *locus in *M. balbisiana*

The MaRGA08 sequence, previously amplified from *M. acuminata *spp. *burmannicoides *Calcutta 4 (A genome) was used to screen a *M. balbisiana *"Pisang Klutuk Wulung" (PKW) BAC library of 36864 clones, representing approximately a nine-fold coverage of the genome [13,33]. Twenty two positive BAC clones were identified, fingerprinted and grouped into two contigs of 14 and 8 BACs, respectively (Additional File 1: MaRGA08 BAC fingerprints and contig assembly). Two BAC clones (MbP026I06 and MbP032N20) were selected for sequencing because they covered most of the first contig and contained all the MaRGA08 hybridization signals. Their sequences showed a 25 kb long identical overlap and were assembled into one contig of 253 366 bp (from hereafter named B1 [EMBL:FN396606]). Based on similar criteria, BAC clone MbP036B13 from the second contig was chosen for sequencing and yielded a 223 694 bp sequence (B2 [EMBL: FN396603]).

The specific features of each sequence are presented in Table 1. Automatic and manually curated sequence annotation predicted 39 and 37 genes and pseudogenes, covering 50.4% and 47.9% (exons and introns, Table 1) of the sequence on B1 and B2, respectively. This corresponds to an overall gene density of one gene per 6.3 kb, similar to the first estimates of gene density in *Musa *[14] although slightly less dense than the average of one gene per 4.2 kb found by [15]. Most of the predicted genes correspond to one large cluster of NB-LRR genes spread over 59% of each sequence in length. This indicates that we have identified two large RGA clusters corresponding to the MaRGA08 probe.

**Table 1:** Features of *M. balbisiana *BAC sequences containing *RGA08 *clusters

	RGA cluster		Flanking sequences	
	B1	B2	B1	B2
Size (bp)	151959	131218	101407	92476
Exons & ψexons (%)	36.1	33.7	18.1	19.1
Introns & ψintrons (%)	13.4	13.3	33.7	30.1
Intergenic (%)	33.6	36.7	42.9	49.9
TE (%)	15.7	15.3	4.4	0
SSR (%)	1.2	0.9	0.9	0.9
Gene & ψgene density (kb/gene)	6.9	6.6	6	5.4
Total predicted gene & ψgene number (RGA & ψRGA)	22 (18)	20 (13)	17	17
ψgene number (ψRGA)	12 (8)	14 (7)	4	4
Number of exon/gene	1	1	5.2	5.7
Number of gene & ψgene on the direct strand	4	3	10	11
Number of gene & ψgene on the complementary strand	18	17	7	6

Percentages of exons and ψexons, introns and ψintrons, TE and SSR were computed according to the size of the nucleotide sequence. Percentage of intergenic sequences represents the rest of the sequence. In these statistics, the five and four Gag-Polymerase -encoding genes of B1 and B2 respectively, were not taken into account. ψ, pseudogene; TE, transposable element; SSR, simple sequence repeat; RGA, resistance gene analog.

### Genetic mapping reveals two haplotypes of the *RGA08 *locus in *M. balbisiana *PKW

**Figure 1:**
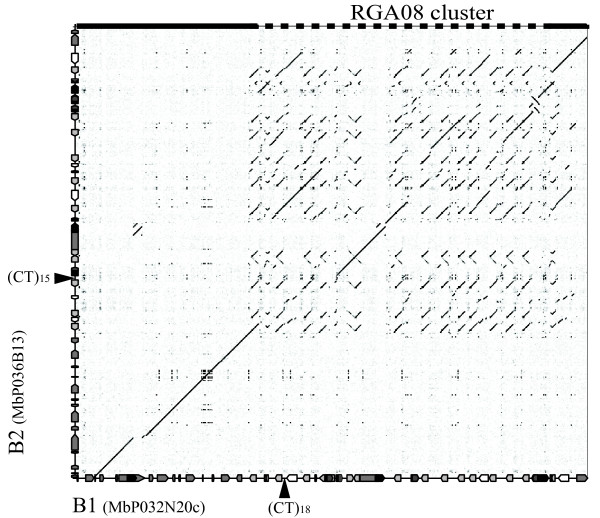
**Dotplot analysis of the *RGA08 *loci in *M. balbisiana***. The dotplot alignment between the B1 and B2 sequences reveals patterns of sequence colinearity and divergence. The X axis corresponds to the MbP026I06-MbP032N20 contig of 253 kb and the Y axis corresponds to 223 kb of BAC MbP036B13. Genes are represented as arrow boxes with the head indicating transcription direction, light grey arrow boxes represent RGAs and black boxes represent transposable elements. Localization of the CT repeat mMaCIR341 microsatellite with the corresponding number of repeats is indicated by arrows. Colinear patterns at the beginning and the end of the contig (highlighted on the top of the figure with a plain line) and complex repetitive pattern (dash line) corresponding to the RGA cluster are indicated.

The B1 and B2 sequences could either correspond to paralogous loci or to orthologous (allelic) regions. The dot plot alignment of the two sequences showed two different patterns: a highly colinear profile at both ends of the sequences and a repetitive and more disrupted pattern in the central part (Figure 1). The repetitive pattern corresponds to the RGA cluster in both sequences. Within this region, colinear relationships are disrupted, although sequence blocks do show colinearity. To test the hypothesis of allelism of the two *RGA08 *loci, a perfect (CT)_n _microsatellite sequence, mMaCIR341, located in the central region (Figure 1), was used as a marker for genetic mapping on an F1 population of 67 triploid hybrids derived from the interspecific cross between the *M. acuminata *autotetraploid cv. IDN110 (4x, AAAA genome) and *M. balbisiana *PKW (BB genome) [34]. The two amplification products from the PKW parent had sizes corresponding to the mMaCIR341 sequences on B1 and B2, while the progeny had either one or the other band but not both and showed a 1:1 Mendelian segregation ratio (χ^2 ^= 0.37) for this marker (Additional File 2: Microsatellite mMaCIR341 segregation profile). This strict segregation of the mMaCIR341 alleles in PKW demonstrates that the B1 and B2 sequences are allelic and represent two haplotypes of the same genetic locus in *M. balbisiana*. FISH analysis of metaphase chromosomes of *M. balbisiana *PKW, using either BAC clone MbP036B13 or the *RGA08 *gene alone as a hybridization probe showed two signals potentially corresponding to one chromosome pair, thus supporting this result (Additional File 3: Fluorescent *in situ *hybridization at the *RGA08 *locus of *M. balbisiana*).

### Conserved and divergent regions between the two *RGA08 *haplotypes

**Figure 2:**
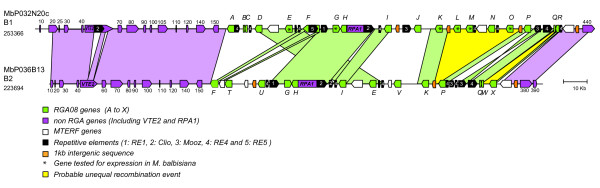
**Genomic organization of the *RGA08 *haplotypes in *M. balbisiana***. *M. balbisiana *B1 and B2 haplotypes corresponding to BAC MbP032N20c and MbP036B13 respectively are represented. Predicted genes, pseudogenes and transposable elements are represented as plain arrowheads with the head indicating the direction of transcription. *RGA08 *genes coding for CC-NB-LRR proteins are indicated as green boxes with a corresponding letter. Genes tested for expression are labelled with a star. White arrows represent *MTERF *genes. Other predicted genes in pink arrows are numbered according to sequence annotation. Repetitive elements are represented as black arrows and numbered according to the list in Table 2. RE4 is a low complexity region with no clear structure. The 1 kb duplicated intergenic sequence is in orange boxes. Simple colinear pattern between haplotypes based on sequence similarity (blast2seq, E-value < = 1e-20) is highlighted for genes and intergenic sequences using light pink trapezoids in the flanking regions of the RGA clusters. Syntenic regions within the RGA clusters are highlighted using light green trapezoids. Identified unequal recombination events are highlighted with yellow trapezoids. The RE3 (Mooz) sequence inverted between haplotypes is indicated with twisted connections.

To investigate the extent of sequence conservation and divergence, we analysed and compared the sequence structure of the two haplotypes. The B1 and B2 sequence overlap corresponds to 245 kb on B1 and 218 kb on B2 with two highly colinear regions flanking the RGA cluster (Figure 1 and Figure 2). The first colinear sequence stretch is 72.5 kb long and contains 10 genes and four pseudogenes. It starts in the middle of a putative aspartate carbamoyltransferase gene which is complete on B1 and only partially covered in B2. Four of the predicted genes have a putative assigned function based on similarity with known proteins in the databases (Additional File 4: Gene list of MbP032N20c and MbP036B13 BACs), six genes encode hypothetical proteins highly conserved with rice predicted proteins and only one gene encodes a hypothetical protein with no corresponding hit in the databases. The remaining three predicted coding sequences (CDS) correspond to fragments of genes. The only interruption in colinearity in this region consists of two indels in predicted introns of the chlorophyl synthase *VTE*2 gene (Figure 2). In B1, the *VTE*2 gene has a 4.4 kb LTR retrotransposon insertion in intron eight and a 0.9 kb *Musa *repetitive sequence in intron 10, both of which are absent from B2. The second highly colinear region located at the end of both sequences is 17.4 kb long and contains two predicted genes coding for a mitochondrial transcription termination factor (MTERF) domain-containing protein and a putative serine/threonine-protein kinase (B1 MbP032N20cg440; B2 MbP036B13g380, Figure 2 and Additional File 4). In both colinear regions, the level of sequence identity between the two haplotypes is very high (99%). Syntenic relationships of B1 and B2 with the rice and sorghum complete genomes were investigated based on best hit (Additional File 4: Gene list of MbP032N20c and MbP036B13 BACs) and phylogenetic relationships (data not shown). A syntenic relationship was predicted within the rice chromosome 6 and its ortholog, the sorghum chromosome 10. This relationship is based on five of the predicted genes and should be confirmed on a larger sequence scale when available. None of the syntenic regions of rice and sorghum contains RGAs.

In between the B1 and B2 colinear regions, the RGA cluster shows a size difference of approximately 21 kb between the two haplotypes. This is not due to a difference in retroelement insertions as their density is similar between haplotypes (Table 1) but rather to a differential expansion of the RGA family (see below). This region also includes a C-terminal fragment of a DNA-directed RNA polymerase I largest subunit *RPA1 *gene and several *MTERF *remnants and pseudogenes interspersed between *RGA08 *genes. Thus, sequence analysis of the two *M. balbisiana *haplotypes showed that they differ from each other by few relatively small indels corresponding to repetitive sequences and in the RGA gene cluster by differential gene expansion.

### Repetitive elements in the *RGA08 *region of *M. balbisiana*

Repetitive sequences are major contributors to genome structure and evolution and are often responsible for intra- and interspecies sequence variation [35,36]. Repetitive sequences identified here include transposable elements and simple sequence repeats (SSR) and represent an overall 10% of each sequence. Transposable elements are mostly present within the RGA clusters rather than in the flanking regions (Table 1). Four types of transposable elements (RE1, Clio (RE2), Mooz (RE3) and RE5, Table 2) have been detected using *ab initio *prediction and similarity based methods. RE1, Mooz and RE5 have a size ranging from 2.8 to 4.2 kb. They show sequence similarity to plant Copia/Ty type of retroelements and have high DNA sequence identity to *Musa *sequences available in databases (Additional File 5: *Musa *RE similarity). Mooz and RE5 have long terminal repeats (LTRs) of a relatively small size (Table 2) and partial or disrupted predicted CDS. RE1 is a remnant copia-like retroelement with a disrupted CDS and no detectable LTRs. An additional repetitive element, Clio, has a 4441 bp size with LTRs of 382 bp and an internal domain with no apparent coding capacity similar to large retrotransposon derivatives (LARD). This element shows several hits to *M. acuminata *and *M. balbisiana *sequences (Additional File 5: *Musa *RE similarity) but not to sequences from other organisms, indicating a new banana specific repetitive element. Target site duplications flanking retroelements insertions have only been found for four elements of the Clio, Mooz and RE5 type and several LTR sequences were partially deleted (Table 2). Truncated Clio and Mooz elements (Table 2) were identified and in addition to the elements described here, fragments of LTR retrotransposon coding sequences are also present on both haplotypes (unlabelled black boxes on Figure 2). This indicates that most of the retroelements have been subject to deletions and sequence rearrangements since their insertion.

**Table 2:** Type, localization and characteristics of repeated elements in the *RGA08 *locus of *M. balbisiana*

Name	Type	ID	Location	Length	5' LTR			3'LTR			TSD	Divergence time
							
			(bp)	(bp)	length	TG	CA	length	TG	CA		(MY)
RE1	Copia-like element	MbP032N20c_te030	129477	2855	-	-	-	-	-	-	-	-
RE1		MbP036B13_te010	100745	2882	-	-	-	-	-	-	-	-

Clio	LARD	MbP032N20c_te010	28466	4441	382	TG	CA	382	TG	CA	ATAC/ATAC	0.294
Clio		MbP032N20c_te040	148954	4441	382	TG	CA	382	TG	CA	GGAG/GGAG	1.189
Clio		MbP036B13_te020	120343	4441	382	TG	CA	382	TG	CA	GGAG/-	
Clio*		MbP036B13_te030	124784	1606	-	-	-	-	-	-	-	

Mooz	Copia-like element	MbP032N20c_te050	166534	3849	115	-	-	112	-	-	-	-
Mooz		MbP032N20c_te060	225945	3346	231	-	-	447	TG	CA	-	-
Mooz		MbP036B13_te050	185390	3688	447	TG	CA	447	TG	CA	CTTTG/CTTTC	2.183
Mooz*		MbP036B13_te040	181339	2464	447	TG	CA	-	-	-	CTTTG/-	

RE5	Copia-like element	MbP032N20c_te020	124026	4301	94	TG	-	171	TG	CA	ACCAC/ACCAC	-

LTR features (length, presence of TG and CA at their ends) and Target Site Duplication (TSD) were determined using LTR_FINDER followed by manual annotation. For complete elements with clearly identified TSD and with LTR length exceeding the empirical limit of 300 bp, divergence time was calculated based on the Kimura-2 parameter distance between LTRs with a substitution rate of 0.9 × 10^-8 ^mutation per site per year. Stars indicate TE fragments. MY, million years.

The position of most retroelements is conserved, with the exception of three of them which contribute to sequence variation between the B1 and B2 haplotypes. A copy of the Clio LARD element in the *VTE*2 gene of B1 is responsible for the first indel polymorphism in the B1 and B2 colinear region and within the *RGA08 *cluster, one Mooz copy is only present on B1 and the RE5 element is only inserted into the B1 *RGA08F-1 *gene (Figure 2).

No class II element (transposon) was identified, but other repeat types were present in this region. In intron 10 of the *VTE2-1 *gene, an indel of 0.9 kb consists of a sequence showing several hits only to *Musa *BAC clones. This sequence is then clearly a banana repeat, although no specific structure was identified. A particularly complex region, RE4 (near RGA08Q, Figure 2) was found highly conserved on B1 and B2. This 4 kb region is AT-rich with 30% of GC compared to the average 40% GC content. It includes three tandem repetitions of 127 bp and a fragment of a copia-type polyprotein. Fragments of the RE4 region ranging from 470 bp up to 1 kb are found at several locations within the *RGA08 *cluster (seven on B1 and five on B2, Figure 2) close to *RGA08 *genes. On B1, adjacent to RE4, three tandem repeats of 162 bp are also present.

In addition, SSRs (*i.e*. dinucleotide up to hexanucleotide repeats) are very abundant, with 112 and 87 SSRs in B1 and B2 respectively. Except for SSR loci that were assigned to RGA coding sequences, and for which it is difficult to find the real ortholog, most of the SSRs are unambiguously found in both haplotypes with their flanking sequences. Of these 75 common non-coding SSRs, 31 are heterozygous, with differences in repeat number varying from one to 46 repetitions.

### A large *RGA08 *gene family

The most divergent region between the B1 and B2 haplotypes corresponds to the *RGA08 *cluster. A total of 31 RGA sequences were identified, differing in number between haplotypes, with 18 present on B1 and 13 on B2 (Table 1). Sixteen are potentially complete genes and the remaining 15 are fragments, pseudogenes or remnants with frameshifts, in frame stop codons and in one case a RE5 insertion (*RGA08F-1*). All the 31 genes belong to the same family as they share 81% of nucleic identity in average (ranging between 65% and 99%). They also share an average of 89% sequence identity with the MaRGA08 probe from *M. acuminata *Calcutta 4. On both haplotypes, only one complete RGA is predicted on the direct strand (*RGA08G-1 *and *RGA08G-2*, Figure 2): it faces an RGA pseudogene on the reverse strand in a tail-to-tail structure (*RGA08H-1 *and *RGA08H-2*) with less than 300 bp between stop codons. Other predicted NB-LRR genes on the direct strand are gene remnants (two on B1 and 1 on B2) all showing the same tail-to-tail structure with another inverted RGA remnant. This indicates a duplication of a region containing this structure followed by loss of sequence. All remaining *RGA08 *genes are predicted on the reverse strand, indicating an expansion of the gene family on this DNA strand. Sequences immediately upstream and downstream the CDS of most *RGA08 *genes are highly conserved, suggesting recent duplication events.

The structural pattern of *MTERF *genes present in the *RGA08 *cluster is also complex. On both haplotypes, one complete copy of this gene with an identical structure of seven exons, is present in the region flanking the RGA cluster (Figure 2). Within the RGA cluster, six *MTERF *pseudogenes and gene remnants are also present, interspersed with *RGA08 *sequences. This indicates common duplication events affecting these sequences and resulting in the complex sequence organisation observed here.

### Conserved allelic relationships between some *RGA08 *homologs

We compared the sequences of the *RGA08 *genes and found that except for one case, the highest levels of sequence identity observed were between RGA pairs from the two haplotypes, thereby defining allelic relationships. The two pairs of RGAs in tail-to tail orientation (*RGA08G-1*/*RGA08H-1 *and *RGA08G-2*/*RGA08H-2*) show 98 to 99% DNA sequence identity and are embedded in a highly conserved syntenic region between the two haplotypes. This region of 26 kb also comprises a highly conserved *RPA1 *C-terminal remnant and is flanked by two repetitive elements RE1 and Clio which are present on both haplotypes (Figure 2). Two other gene pairs (*RGA08K-1 *and *RGA08K-2*, *RGA08P-1 *and *RGA08P-2*) together with gene remnants *RGA08Q-1*/*RGA08Q-2 *showed 99% sequence identity and therefore also correspond to allelic pairs. The highest level of sequence identity between pairs was always between genes from different haplotypes, with one exception: *RGA08I-1 *and *RGA08I-2 *showed 98% sequence identity to each other as well as to *RGA08D-1*, suggesting a recent duplication of this gene in the B1 haplotype. The pair *RGA08D-1 *and *RGA08I-2 *is present within a highly conserved region between haplotypes which comprises the *MTERF1 *pseudogene and the *RGA08E-1*/*RGA08E-2 *pair (95% sequence identity). This sequence block is in a different position in B1 compared to B2 (Figure 2) due to sequence rearrangements within the *RGA08 *cluster. Finally, *RGA08F-1 *and *RGA08S-2 *share 95% identity in their CDS but they are not in syntenic position and they also differ by the RE5 LTR retroelement insertion in *RGA08F-1 *which interrupts its coding sequence (Figure 2). No clear allelic relationships could be found for the remaining RGAs which are therefore considered as paralogs.

### Two clades of *RGA08 *genes are present at the *RGA08 *locus

**Figure 3:**
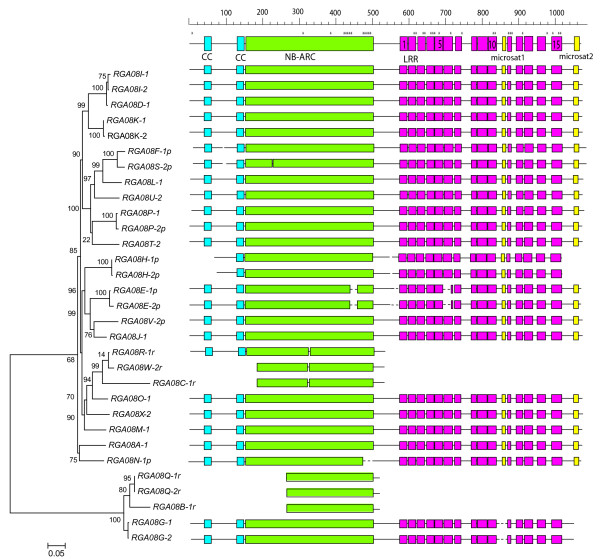
**Phylogenetic relationships of *RGA08 *predicted CDS and schematic representation of the corresponding CC-NB-LRR domains**. Different colored boxes indicate the RGA protein domains identified manually after a multiple *RGA08 *CDS alignment. Blue boxes represent the N-terminal coiled-coil region (CC). Green boxes represent the nucleotide-binding region and the ARC domain shared by APAF-1, R and CED-4 proteins (NB-ARC domain). Pink boxes represent the 15 leucine-rich repeats (LRR). Yellow boxes represent simple sequence repeats. Sites predicted to be under positive selection according to PAML analysis are indicated by stars. The sizes of boxes and gaps between domains are drawn according to scale.

The *RGA08 *genes encode predicted proteins with two coiled-coil predicted motifs (Additional File 6: Prediction of coiled-coil motifs in RGA08) at the N-terminal followed by an NB-ARC domain with all known conserved motifs (P-loop, RNBS-A, kinase-2, RNBS-B, RNBS-C, GLPL, RNBS-D, MHD [17]) and a C-terminal leucine-rich repeat domain with 15 LRR repeats for the complete genes. No intron was predicted which is consistent with genome wide studies in Arabidopsis, grapevine and poplar which indicate that CC-NB-LRR genes are generally encoded by a single exon [17,37]. Phylogenetic analysis identified two clades of *RGA08 *genes (Figure 3). The first comprises all RGAs predicted on the direct strand of both haplotypes, namely the two complete genes of 3147 bp (*RGA08G-1 *and *RGA08G-2*) and three remnants of 711 to 720 bp covering the NB region (*RGA08B-1*, *RGA08Q-1 *and *RGA08Q-2*). These remnants are more similar to each other than to the complete *RGA08G-1 *and *RGA08G-2*, which indicates that at least two duplicated genes of this clade were originally present in this region. The second clade comprises all RGAs predicted on the reverse DNA strand. These genes have an average size of 3.2 kb (from 2793 to 3310 bp). Three remnants of these genes covering the NB region have a size of 1026 to 1555 bp. A 70% level of DNA sequence identity was observed between clade one and clade two genes and their overall structure is highly conserved (Figure 3). One major difference between the two clades is the presence of coding microsatellite sequences only in the LRR region of clade 2 *RGA08 *genes. The first microsatellite (TTC trinucleotide repeat) between LRR10 and LRR11 results in a polyserine tract which varies from three up to 16 amino acids. The second microsatellite is a compound (GAT)n(GCT)n(GAA)n motive at the 3'end of group 2 RGA genes. The GAA triplet show the largest size expansion (from four up to 21 repeats) resulting in a predicted acidic coiled-coil domain (polyE, aspartic acid) for genes showing a high number of repeats.

### Evolution of the *RGA08 *cluster: intragenic and intergenic recombination

Sequence exchange was previously shown to be a main mechanism of *R *gene evolution [38,39]. Using the program RDP3.34 which integrates several modules for detection of recombination and gene conversion events, we investigated the degree of sequence exchange between the *RGA08 *homologs. A total of 21 events (at p < 0.05) were detected between *RGA08 *genes. These sequence exchanges affected all *RGA08 *genes except for the genes in tail-to-tail structure (*RGA08G-1*/*RGA08H-1 *and *RGA08G-2*/*RGA08H-2*).

**Figure 4:**
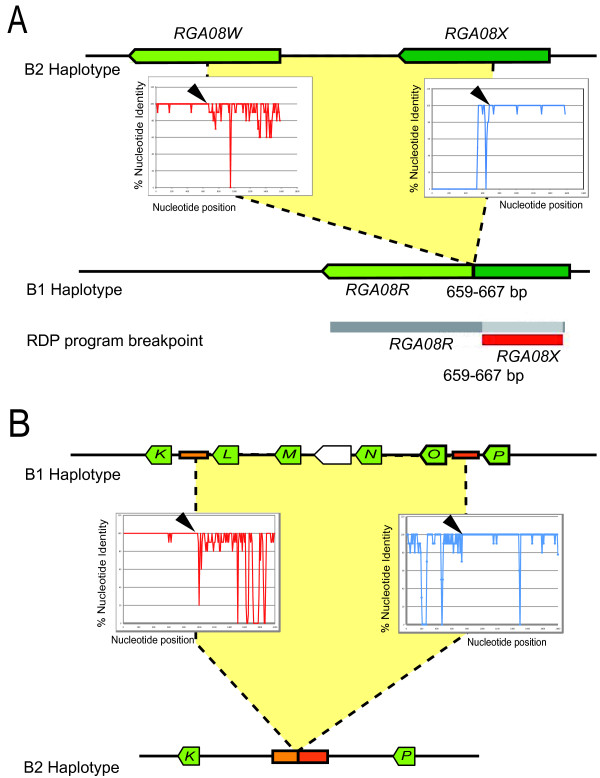
**Unequal recombination breakpoints within the *RGA08 *gene cluster**. (A) An intragenic unequal recombination event between *RGA08X *and *RGA08W *results in the actual structure of the *RGA08R *sequence. The RDP software detected the region of sequence identity between *RGA08X *and *RGA08R *and indicated a recombination breakpoint on *RGA08X*. Manual inspection of *RGA08 *sequence alignments indicated *RGA08W *and *RGA08X *as likely parental sequences. The percent nucleotide identity of *RGA08X *(red curve) and *RGA08W *(blue curve) is plotted along the sequence of *RGA08R*. A shift in sequence identity levels (black arrows) is observed indicating a putative recombination event. (B) Unequal recombination mediated by duplicated 1 kb intergenic sequences (orange boxes). Intergenic sequences between *RGA08K *- *RGA08L *and *RGA08O-RGA08P *in B1 (top) have recombined to produce the actual intergenic sequence structure between *RGA08K - RGA08P *in B2 (bottom). Percent of nucleotide identity between 1 kb intergenic sequences of B1 is plotted along the B2 sequence. High sequence identity is visible at the beginning of the *RGA08K *- *RGA08L *intergenic sequence (red curve) and at the end of the *RGA08O-RGA08P *intergenic sequence (blue curve). Black arrows indicate breakpoint position.

Unequal recombination resulting in increase or decrease in RGA numbers in one or the other haplotype also takes place in the *RGA08 *cluster. One intragenic recombination event was detected by the RDP program based on conserved sequence blocks between *RGA08R *and *RGA08X *(Figure 4A). Manual inspection of the alignment of *RGA08R*, *RGA08X *and *RGA08W *suggests that the actual structure of the *RGA08R *remnant results from an unequal recombination event between *RGA08W *and *RGA08X *sequences likely resulting in the elimination of *RGA08X *from B1. Other recombination events detected by RDP could not lead to clear reconstruction of structural changes affecting the *RGA08 *cluster. We therefore manually examined colinearity breakpoints between haplotypes within the *RGA08 *cluster. In addition to the *MTERF *coding sequences present in the *RGA08 *cluster, a 1 kb intergenic sequence present at 2 kb upstream the start codon of the complete *MTERF *gene is duplicated and conserved (70 to 98% identity) within the RGA cluster at three and two additional locations on B1 and B2 respectively (orange boxes on Figure 2). Two of these 1 kb long sequences are flanking the region carrying the *RGA08L *up to *RGA08O *genes on B1 (Figure 2). Another copy is present between the *RGA08K *and *RGA08P *genes on B2. Sequence alignment of these regions identified a shift of sequence identity levels between B1 and B2 within this intergenic sequence (Figure 4B). This suggests that the absence of the *RGA08L *to *RGA08O *region from the B2 halotype could be due to an intergenic unequal recombination event between duplicated non-coding sequences.

### Diversifying selection

Sequence exchange is a major mechanism of RGA evolution but its possible homogenizing effect is counteracted by diversifying selection acting on specific residues of CC-NB-LRR proteins [38]. We investigated the sites under positive selection in the CC-NB region and the LRR region of complete genes using the PAML package [40]. Two likelihood-ratio tests (M1a against M2a and M7 against M8) indicated that sites were under diversifying selection for the CC-NB (M7/M8, χ^2 ^= 145, P < 0.001) and LRR data sets (M7/M8, χ^2 ^= 246, P < 0.001). The majority of positively selected sites (21 out of 40) were located in LRRs (Figure 3) and 14 of them were solvent-exposed residues (x residues in the LxxLxLxx motif) which are predicted to be involved in effectors' recognition in R proteins.

### Expression of *RGA08 *genes

Six specific primer pairs were defined in divergent parts of 3'-untranslated regions to test whether the corresponding *RGA08 *genes (*i.e. RGA08E-1*, *RGA08G-1*, *RGA08K-1*, *RGA08L-1*, *RGA08M-1*, *RGA08O-1*, Figure 2) are expressed *in planta*. All tested genes were found expressed in different tissues of *M. balbisiana *(Additional file 7: Real-time qPCR analysis of RGA08 expression) indicating that several members of the *RGA08 *cluster are expressed and are thus potentially functional.

### Divergence time of *M. balbisiana *haplotypes

To estimate the timing of evolution of the two *M. balbisiana *haplotypes, we calculated the extent of sequence divergence between the conserved colinear sequences on both sides of the RGA cluster. In addition to indels, these sequences differ by single nucleotide polymorphisms with an overall nucleotide diversity of 0.95%. Twelve complete predicted allelic pairs have been used to calculate the ratio of synonymous mutation per synonymous site according to [41]. On the 12.9 kb of coding sequences (Additional File 8: Estimation of divergence time between PKW haplotypes), 27 synonymous mutations were detected giving a p-distance of 0.0088. This is lower than the value found between genes of *M. acuminata *and *M. balbisiana *(ks = 0.04) which diverged 4.6 MYA. Using the substitution rate that was recently determined for banana coding sequences (0.45 × 10^-8 ^mutations per site per year; [15]), a divergence time of almost 1 MYA was obtained for *M. balbisiana *PKW haplotypes.

We have also calculated insertion times of complete retroelements with TSDs and LTR sequences above 300 bp in length using the method of [42]. An evolution rate double than the one determined for coding sequences was used taking into account the more rapid evolution of noncoding sequences [36]. Based on this, the Clio element located into the *VTE2-1 *gene has inserted 0.3 MYA (Table 2). This value indicates recent activities of LTR retrotransposons in the *M. balbisiana *genome and is coherent with an insertion of Clio into haplotype B1 rather than elimination from B2. A second Clio element in syntenic position in the two haplotypes has inserted in B1 around 1.2 MYA and thus possibly predates haplotype divergence. Its insertion time could not be confirmed in B2 due to rearrangements. Mooz elements located on the left side of the *RGA08 *cluster are also in syntenic position but their structure is complex with an inversion and indels. However, the complete Mooz element in B2 has inserted 2.2 MYA confirming presence of Mooz in the ancestral haplotype.

## Discussion

### Sequence divergence of *M. balbisiana *haplotypes

Despite the increasing number of genomic resources developed for *Musa *species, no study has addressed the question of haplotype divergence at the sequence level. Molecular marker analyses have uncovered a large diversity among *M. acuminata *subspecies and also to some extent in *M. balbisiana *[43,44]. *Musa *wild species such as *M. balbisiana *are generally outcrossing even if inbreeding is not totally excluded, due to potential crosses between vegetative offspring. We have identified two sequence haplotypes in wild *M. balbisiana *PKW in the region of the *RGA08 *cluster which allowed us to assess their degree of sequence divergence. The structure and divergence of sequence haplotypes have been studied in different plant species including grasses such as barley, wheat and maize and recently in poplar and sugarbeet. They revealed a mosaic structure of conserved and non-conserved sequences at orthologous or allelic regions [35,45-48]. The differences mostly affected the intergenic space and were based on repetitive element insertions and indels of various sizes likely due to unequal homologous recombination and illegitimate recombination events. In maize, intraspecific comparisons have additionally showed a difference in gene content between inbred lines [49]. Here, two regions of contrasting features were identified: the divergent *RGA08 *gene cluster and the highly conserved non-RGA region. In the latter, the gene content was fully conserved and the intergenic regions were also highly conserved. The two relatively small indels correspond to 5.8% of the colinear sequence indicating few rearrangements in the region flanking the *RGA08 *cluster. In rice, indels account for 13% of genome divergence between the indica and japonica subspecies [36], whereas in poplar, indel rate was found to vary from 0.5 to 14.8% of non aligning segments between four regions of poplar haplotypes [47]. In terms of nucleotide diversity, our values (0.95% based on the complete colinear region and 0.34% for coding sequences) are very similar to those in rice subspecies in overall substitution rate (0.96%) and for genic regions (0.25% for coding sequences) [36] and are lower than those observed in maize (0.8% in coding sequences [50]) or sugarbeet (5.14% overall, 0.96% for coding sequences [48]). Haplotype divergence rates might vary from one region of the genome to the other as shown in poplar [47] where sequence diversity varied from 0.32 to 1% in 320 kb spread over four compared regions. We calculated the nucleotide divergence at a second *M. balbisiana *PKW locus (EPRV locus, [51]) and found there a value of 0.25% overall nucleotide divergence (0.03% for coding sequences). Thus, the degree of polymorphism observed between these two haplotypes of *M. balbisiana *is in the range of what was observed for intraspecies diversity in rice and in poplar but is lower than that found in maize and sugarbeet. This degree of polymorphism is compatible with the recent divergence time of 1MY we calculated for these *M. balbisiana *haplotypes.

### The role of repetitive elements

The repetitive fraction of plant genomes is highly variable and can constitute more than 50% of the genome landscape as in some grass species [52]. In Musa, a proportion of 35% of repetitive sequences was estimated based on BAC end sequences of *M. acuminata *Calcutta 4 [14]. The sequences analysed here showed a relatively low repeat content of 10% in size. The proportion of identified retroelements in the overall *RGA08 *region corresponds to the average of 2.6 retrotransposons per 100 kb found in gene-rich *Musa *BAC clones [15] although this value is possibly underestimated as many *Musa *transposable elements are still unknown.

Transposable elements identified here all belong to class I LTR retrotransposons. They showed an uneven distribution, with the majority present within the RGA gene cluster. It is difficult to know, however, if this difference in repeat distribution is a cause or a consequence of the *RGA08 *gene amplification process. Estimation of insertion times of RE2/Clio and RE3/Mooz retroelements (0.3 to 2.2 MY) indicates recent insertions after the divergence of the *Musa *A and B genomes, 4.5 MY ago. The presence of older remnants of LTR retrotransposons and the fact that most elements identified here are disrupted in their sequences is consistent with mechanisms of deletion affecting repetitive elements as previously described in other genomes such as rice and Arabidopsis [53,54]. The recent RE2/Clio insertion (0.3 MYA) contributed to haplotype divergence in the non-RGA colinear region and reflects very recent activity of this retrotransposon family in *Musa*. Indel sequence variation between haplotypes was due to repetitive sequences and suggests a major role of repeats in the divergence of gene-rich regions of the Musa genome.

Sequences presented here are particularly rich in SSRs, when compared to available banana BAC end sequences in which only one SSR was observed every 13 kb [14]. Density of SSRs in this locus is much higher, with an average of one every 1.5 kb. A strong bias exists within the RGA cluster, with one SSR every 1.1 Kb compared to the flanking sequences with one SSR every 2.6 kb. The high density of SSRs within the RGA cluster is due, at least partly to the presence of coding microsatellites within *RGA08 *genes.

### *RGA08 *cluster evolution in *M. balbisiana*: the impact of duplicated intergenic sequences

The *RGA08 *cluster is a member of the large *R *gene clusters identified in plant genomes, with 13 and 18 CDS of the same gene family in less than 152 kb on each haplotype. Large *R *gene clusters have been identified in several species such as common bean [26], at the tomato *I2 *locus (7 genes in 90 kb, [55]) and in lettuce where the *RGC2 *gene family comprises 32 members within 3 Mb [39]. Within the *RGA08 *cluster, drastic changes have disrupted colinearity between haplotypes. These include a difference in *RGA08 *gene number, in repetitive elements and a reshuffling of whole sequence blocks carrying *RGA08 *genes and fragments of *MTERF *genes. Such features are similar to what was previously described in other plants at *R *gene loci (*e.g*. [22]). One specific feature here is the high level of sequence conservation of both genic (92% identity in average) and intergenic (average 78% identity) sequences between clade 2 paralogs which suggests recent duplication events.

Two clades of genes coexist on the *RGA08 *cluster with one clade showing a particularly large expansion on one DNA strand. The two types of *RGA08 *genes likely originated from the same ancestral sequence and followed two different evolutionary paths. The actual structure of the cluster and the presence of remnants in tail-to-tail orientation suggest a possible model of evolution where a duplication of the ancestral *RGA08 *sequence was followed by an inversion and a differential expansion on one DNA strand. The structure in tail-to-tail orientation was also likely duplicated then partially deleted. It was frequently reported that repetitive elements are associated to *R *gene clusters [27,56] and the presence of duplicated sequences such as the LTRs of LTR retrotransposons could provide templates for unequal recombination thus generating diversity at *R *gene clusters. Despite the identification of several LTR retrotransposons, we could not find evidence for their involvement in unequal recombination events. This is likely due to the fact that the complete retroelements we identified are not highly duplicated within the cluster. One particular duplicated sequence, the RE4 region contains remnants of a copia-like polyprotein. Fragments of RE4 have been found close to duplicated *RGA08 *genes and although we could not demonstrate such events, it is not excluded that this particular low complexity region corresponds to an old repetitive sequence involved in the duplication process of the *RGA08 *clade 2 genes. In addition to RE4, three types of sequences are found highly duplicated within the *RGA08 *cluster: the *RGA08 *genes, fragments of the *MTERF *gene and intergenic non-coding sequences of varying sizes which were found conserved at different positions. We could show that sequence exchange occurred between *RGA08 *sequences. In addition, we found strong indication that a duplicated non-coding sequence with no specific features was likely involved in an intergenic unequal recombination event resulting in a large difference in *RGA08 *gene content between haplotypes. The *RGA08 *gene family thus likely evolved by mechanisms involving intragenic and intergenic unequal recombination, resulting in a difference in size and *RGA08 *gene content between haplotypes. In spite of this, allelic relationships have been retained for some of the *RGA08 *genes.

A particular feature of the *RGA08 *family is the differential expansion of coding microsatellites in the LRR region encoding a polyserine and a poly-glutamic acid motif. Such a feature has been described for the *RGC2 *gene family in lettuce [57] and the *Hero *nematode resistance gene in tomato [58]. Amongst *Hero *paralogs, the functional gene showed the largest expansion of the microsatellite. Currently the function of these motifs is unknown, although their presence in *R *genes and RGAs across different species suggests a potential functional impact of such regions.

The *RGA08 *genes show all typical modes of evolution of *R *gene clusters. Several of them are expressed, encode potentially complete proteins, and positive selection has been identified on specific residues mostly in LRR repeats. Such a large gene cluster with a likely recent expansion could encode functional resistance genes. Unfortunately, no resistance phenotype has yet been mapped in *Musa *species. Markers derived from the different *RGA08 *genes here identified could help in targeting a specific disease resistance trait in *Musa *species.

## Conclusions

The comparative analysis of the sequence structure of two recently diverged *M. balbisiana *haplotypes revealed an overall high level of sequence conservation of gene-rich regions but also identified recent activity of repetitive elements contributing to sequence divergence. In addition, our analysis of the sequence organisation of the *RGA08 *locus in wild banana has shown that within one diploid genotype, considerable variation exists in the number, sequence organisation, sequence polymorphism and mode of evolution of RGA genes. Larger scale studies on different *M. balbisiana *genotypes are required to better apprehend the haplotype divergence and evolution in this species and help breeders in managing the integration of *M. balbisiana *in breeding programs. In addition, the whole genome sequence of *M. acuminata *(A genome, http://www.cns.fr/spip/September-8th-2009-Banana-genome.html) will soon become available which will allow further comparative studies of the different Musa genomes.

## Methods

### BAC library screening, contig construction and BAC sequencing

BAC library screening and hybridization were performed as described by [33]. A contig construction was performed manually based on MaRGA08 hybridization profiles. Contigs were subsequently confirmed by FPC reconstruction based on *Hin*dIII fingerprints using the following parameters: tolerance, 7; cutoff, 10e-12. BACs were sequenced at 15 fold coverage and assembled using Phred-Phrap. Automatically generated draft sequences were manually curated using CONSED viewer. To ensure correct sequence assembly, *in silico *fingerprints were compared to BAC DNA fingerprints on pulse field gel electrophoresis using several restriction endonucleases (*Hin*dIII, *Eco*RI, *Swa*I, *Sfo*I and *BssH*II).

### Segregation analysis

Genetic mapping was performed on a population of 67 allotriploid hybrids (AAB) previously described in [34]. This population is derived from interspecific hybridization of autotetraploid *M. acuminata *(IDN110 4x, AAAA) and diploid *M. balbisiana *Pisang Klutuk Wulung (PKW). The microsatellite mMaCIR341 was amplified by PCR with primer pair mMaCIR341-F (5'-TGAAGGAATCATCAAGCACAA-3)' and mMaCIR341-R (5'- GGGAAAAATTCAGCACTTGA-3)' using 25 ng of plant DNA or 2 ng of BAC DNA as template. PCR conditions were those described for banana SSRs [59]. The ^33^P-labelled amplification products were resolved on polyacrylamide gels. Dried gels were exposed overnight and autoradiographed using a STORM 820 scanner (Amersham). Segregation data were scored manually.

### Fluorescent *in situ *hybridization (FISH)

FISH experiments were performed on root tips of greenhouse grown *M. balbisiana *PKW as described in [60]. MbP036B13 BAC DNA or PCR amplified *RGA08 *gene were labelled with biotin using BioPrime DNA labelling system (Invitrogen) and detected with Texas Red. The hybridization was performed with 100ng of probe as described in [60]. The chromosomes were counterstained with DAPI (4'.6-diamino-2-phenylindole). Images were taken from a Leica DMRXA2 epifluorescence microscope equipped with a cooled Hamamatsu Orca AG camera and Volocity acquisition software (Perkin Elmer).

### Gene model prediction, sequence alignment and comparison

Gene structures were predicted using the EuGène combiner release 3.2 [61] with rice-specific parameters that integrate several evidences. Gene models were predicted with *ab initio *gene finders, EuGèneIMM and Fgenesh [62]. Translation starts and splice sites were predicted by SpliceMachine [63]. Available monocotyledon ESTs from EMBL were aligned on the genome using Sim4 [64]. Similarities to protein sequences were identified using BLASTx (NCBI-BLASTALL) [65] on UniProt [66]. Polypeptide functions were also predicted by integrating several evidences. Protein similarities were searched using tBLASTn on translated monocotyledon ESTs and BLASTp on UniProt. Protein domains were predicted with InterproScan [67]. Clusters of orthologous genes between the predicted polypeptides and the proteomes of *O. sativa *(TIGR release 5.0) and *S. bicolor *(JGI release 1.0) were also identified using the pipeline GreenPhyl [68]. The predicted genes were manually annotated using Artemis [69]. A gene is considered complete if its coding sequence (CDS) is canonical and matches significantly a known sequence in the public databanks with coverage parameters Qcov and Scov greater or equal to 0.8. Under these parameters, the CDS is also predicted to be functional. If a gene contains mutations that could prevent correct expression (*i.e*. missing start codon or stop codon, non canonical splicing site, frameshift or in frame stop codon), it was considered as a pseudogene. A polypeptide was annotated as a fragment if its coverage (Qcov) is inferior to 0.8 when comparing its length to the length of the match with the best significant hit. We annotated a gene as remnant if it is composed of a small fragment (Qcov <0.3), more than three fragments (Qcov <0.5) and/or if it has more than two mutations preventing correct CDS expression. BAC sequence annotation is available on a genome browser http://gnpannot.musagenomics.org/cgi-bin/gbrowse/musa/. Sequence comparison between haplotypes was performed using dotplot analysis [70]. Local alignments were performed using BLASTn [65] and visualised with the Artemis Comparison Tool (ACT) [69].

### Identification and characterization of *Musa *repeats

Consensus of repetitive and transposable elements (RE, TE) were first defined *ab initio *from the 66 *Musa *BACs available in the databases, using the TEdenovo pipeline of the REPET framework as described in [71]. Then, repeats were predicted using an improved version of the TEannot pipeline described in [72]. The candidate regions for the presence of repeated elements on MbP036B13 and MbP032N20c were subsequently analysed with LTR_FINDER [73]. TEs of MbP036B13 BAC and MbP032N20c were then manually annotated using Artemis [69] and the presence of LTRs was checked using dotplot analysis of the sequence against itself. Classification of characterized transposable elements was performed based on BLASTx against REPET edition of Repbase (repbase1302_aaSeq_cleaned_TE.fa). Insertion time of LTR retroelements was calculated for complete elements according to the formula T = K/(2r) where T is the time of divergence, K is the number of base substitution per site between LTR sequences, and r is the substitution rate [42]. Nucleotide substitutions were calculated using MEGA release 4 [74] with the Kimura 2-parameter substitution model. A rate of 9.10^-9 ^mutations per site per year was used. This rate was 2-fold higher than that determined for coding sequences in banana based on the assumption that non-coding sequences evolve more rapidly [36].

### Divergence time of *M. balbisiana *allelic regions

Divergence time between two homologous protein-coding sequences was calculated according to the formula T = p_S_/(2r) where T is the time of divergence, p_S _the number of synonymous mutations per synonymous sites between the two sequences and r the synonymous substitution rate. p_S _was determined using the Nei and Gojobori method in MEGA4 [74]. The synonymous substitution rate of 4.5 per 10 ^9 ^years determined for banana coding sequences [15] was used.

### Analysis of the CC-NB-LRR genes

The 31 CC-NB-LRR coding nucleotide sequences were aligned with MAFFT [75] and checked manually to verify that codon alignment corresponds to amino acid alignment (Additional File 9: Sequence alignment of the coding sequences of *RGA08 *genes). The maximum-likelihood tree was computed using aLRT-PhyML [76][77] under the HKY85 substitution model. Instead of the classical Felsenstein's bootstrap, the approximate likelihood-ratio test (aLRT) non-parametric branch support based on a Shimodaira-Hasegawa-like procedure (SH-like branch support) was used.

The conserved motifs of NB-LRR proteins (P-loop, RNBS-A, kinase-2, RNBS-B, RNBS-C, GLPL, RNBS-D, MHD) were identified based on [17]. LRR repeats were defined manually based on the consensus LxxLxxLxxLxLxx(N/C/T)x(x)LxxIPxx where L is an aliphatic amino acid and x is any amino acid [78]. Coiled-coil structure was predicted by COILS http://www.ch.embnet.org/software/COILS_form.html. Sequence exchange was evaluated using the programs RDP, Geneconv [79] and Bootscan [80], implemented in RDP version 3.34 [44] with defaults parameters except for the Bootscan method where 200 bootstraps and a 90% cut-off value were used.

For the detection of positive selection, sites under diversifying selection were investigated with the maximum likelihood program CODEML of the PAML package [81]. Sixteen complete genes were analyzed: ten genes for the B1 haplotype (*RGA08A, RGA08D, RGA08G-1, RGA08I-1, RGA08J, RGA08K-1, RGA08L, RGA08M, RGA08O and RGA08P*) and six genes for the B2 haplotype (*RGA08T, RGA08U, RGA08G-2, RGA08I-2, RGA08K-2 and RGA08X*). As suggested by [17], the alignment of the coding sequences was cut into two parts: the CC-NB and the LRR, 30 amino acids beyond the MHDL motif. The two trees were computed using PhyML. Two LRTs, M2a against M2a and M7 against M8, were run for testing positive selection.

### Expression analysis

Two micrograms of total RNA were extracted from different PKW tissues (fruit, flower, root, stem, and leaves) using the hot borate method as described in [82]. RNA was treated with RQ1 DNase (Promega, Charbonnières, France) to remove possible contaminating genomic DNA. First-strand cDNA was synthesized using AMV reverse transcriptase enzymes (Promega, Charbonnières, France) and random hexamers according to the manufacturer's instructions. Quantitative Real-Time PCR was performed to analyse mRNA accumulation using the ABI PRISM 7000 sequence detection system (Applied Biosystems, Courtaboeuf, France). All qPCR experiments were performed as described by [83]. Six RGAs (*i.e. RGA08E-1, RGA08G-1, RGA08K-1, RGA08L, RGA08M, RGA08O*) were selected for expression analysis. Specific primer pairs were designed in the predicted 3'UTR of each selected gene. The specificity of PCR amplification was examined by monitoring the dissociation curves during qPCR reactions. The relative fold differences in expression of each gene between samples were determined using the 2-ΔΔCt formula [84]. For each gene and sample, analysis was performed in triplicate on two independent RNA extracts. The MaACT (actin, EF672732) gene was used as internal control to standardise the difference between template amounts and expression level in the peel of green fruit was used as calibrator.

## Accession numbers

Sequences presented in this paper are available under the following accession numbers in the International Nucleotide Sequence Databases (INSD): FN396603; *M. balbisiana *clone BAC MBP-36B13, complete sequence; FN396606; *M. balbisiana *BAC contig MBP-32N20c, complete sequence.

## Authors' contributions

All authors read and approved the final manuscript. FCB: participated in the study design, performed BAC physical mapping and genetic analysis, participated in BAC annotation and sequence analysis and jointly wrote the manuscript. SB: participated in the study design, coordinated BAC annotation and sequence analysis, performed selection tests and jointly wrote the manuscript. MR: performed *Musa *- Sorghum synteny analysis. TM: performed BAC sequencing and sequence assembly. RNGM: participated in the study design and performed BAC selection. MRG: designed and performed FISH experiments. DMAM: designed and performed gene expression experiments. NY: participated in the study design, coordinated the study, analysed BAC sequences and jointly wrote the manuscript.

## Supplementary Material

Additional file 1 **MaRGA08 BAC fingerprints and contig assembly**. A - *Eco*RI fingerprint of positive *Musa babisiana *BAC clones hybridized with the MaRGA08 probe. Lanes 1 to 22 correspond to BAC clones MbP004L16, MbP004M06, MbP012B09, MbP014P10, MbP015E06, MbP017K14, MbP019H11, MbP022M12, MbP025J05, MbP026I06, MbP027C10, MbP032E10, MbP032N20, MbP035J24, MbP036B13, MbP046G13, MbP053I03, MbP055C19, MbP056J15, MbP056M16, MbP086F08, MbP090E06, respectively. Lane M: 1 Kb ladder DNA marker. Sizes in Kb are indicated on the left. Black-white arrows indicate BAC contig groups. B - Resulting manually constructed contigs. BACs are represented by horizontal lines, circles represent hybridization bands in figure 1A. Bands of similar size are joined by vertical lines. BACs with all MaRGA08 hybridizations signals were chosen for sequencing (highlighted with black circles). Click here for file

Additional file 2 **Microsatellite mMaCIR341 segregation profile**. The autoradiography of mMaCIR341 SSR amplification on the whole F1 mapping population from the cross PKW (BB) X IDN4x (AAAA) is presented. Controls are loaded on both sides of the gel: Parent PKW, lanes 1 and 76; BAC MbP036B13 (B2) lanes 2 and 77, BAC MbP026I06 (B1), lane 3 and 78; Parent IDN4x AAAA, lanes 4 and 79. Of the 71 individuals of the AAB F1 progeny, four have missing data (lanes 13, 24, 39, and 68), the remaining 67 exhibit one mMaCIR341 allele or the other demonstrating strict segregation of mMaCIR341 alleles at this locus. mMaCIR341 allele sizes are indicated on the left side. Click here for file

Additional file 3 **Fluorescent *in situ *hybridization at the *RGA08 *locus of *M. balbisiana***. *In situ *FISH of metaphase spread chromosomes of *M. balbisiana *PKW using Biotin/Texas Red labelled BAC MbP036B13 (A) and 1.4 kb of MaRGA08 gene (B). Arrows indicate the two labelled chromosomes. Click here for file

Additional file 4 **Gene list of MbP032N20c and MbP036B13 BAC sequences**. The MbP032N20c and MbP036B13 polypeptides were aligned with BLASTALL to the rice proteome (MSU Annotation Release 6.0) to determine best hit relationships (BH). Rice genes used to infer syntenic relationships are in red. C, complete; Ψ pseudogene; f, fragment; N-f, N-terminal fragment;C-f, C-terminal fragment; r, remnant; N-r, N-terminal remnant; C-r, C-terminal remnant. Click here for file

Additional file 5 ***Musa *repetitive element (RE) similarity**. Blastn hits were reported if the query coverage >50%. Click here for file

Additional file 6 **Prediction of coiled-coil motifs in RGA08 predicted proteins**. The probability of forming stable coiled-coils predicted by the COILS program http://www.ch.embnet.org/software/COILS_form.html is plotted against the amino acid residues of RGA08K-1. Predicted coiled-coil structures correspond to residues 34-54 and 123-143. Click here for file

Additional file 7 **Real-time qPCR analysis of RGA08 expression**. Quantitative Real-Time PCR was used to analyse the mRNA accumulation of: RGA08E, RGA08G, RGA08K, RGA08L, RGA08M (**A**) and RGA08O (**B**) in different tissues of PKW including peel of mature green fruit (Gpe), pulp of mature green fruit (Gpu), peel of ripe fruit (Rpe), pulp of ripe fruit (Rpu), flower (Fw), bract (Bc), root (Rt), seed (Se), stem (St), young leaf (Yl) old leaf (Ol). The y axis represents the relative fold difference of mRNA level and was calculated using the 2-ΔΔCt formula with actin as reference. The mRNA fold difference was relative to peel tissue of green fruit used as calibrator. Each data point is the mean of values obtained from qPCR reaction performed in triplicate on one sample. Each sample was prepared from tissues originated from two replicate plants or bunches. Vertical bars indicate standard deviation (S.D.). When no bar is shown, S.D. was smaller than the symbol. Primer pairs used in this study are indicated (**C**). Click here for file

Additional file 8 **Estimation of divergence time between PKW haplotypes**. Gene names are those listed in Additional File 4, sequence length is given in base pairs, Sd is the number of synonymous mutations, p-distance is estimated as the ratio of synonymous mutations per synonymous sites [41] and divergence time is calculated using a synonymous substitution rate of 0.45 per 10^9 ^year [15]. Click here for file

Additional file 9 **Sequence alignment of the coding sequences of *RGA08 *genes**. Phylip format Aligned coding sequences for all members of the RGA08 family are provided in phylip format. Click here for file
