# Supplementary figures and images for: Mechanisms of haplotype divergence at the RGA08 nucleotide-binding leucine-rich repeat gene locus in wild banana (Musa balbisiana)
Source: BMC Plant Biol. 2010 Jul 16;10:149. doi: 10.1186/1471-2229-10-149 (PMC3017797; doi:10.1186/1471-2229-10-149)

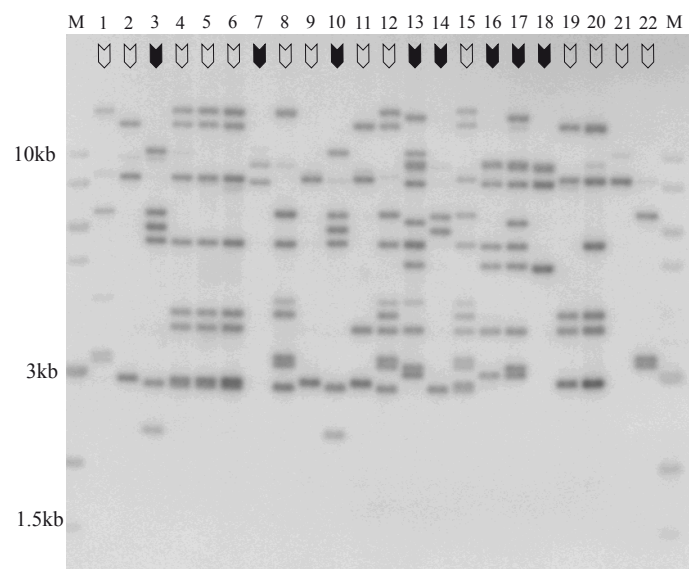

A

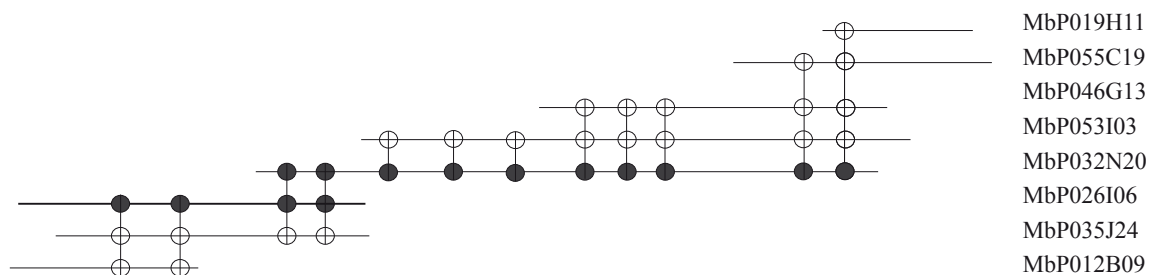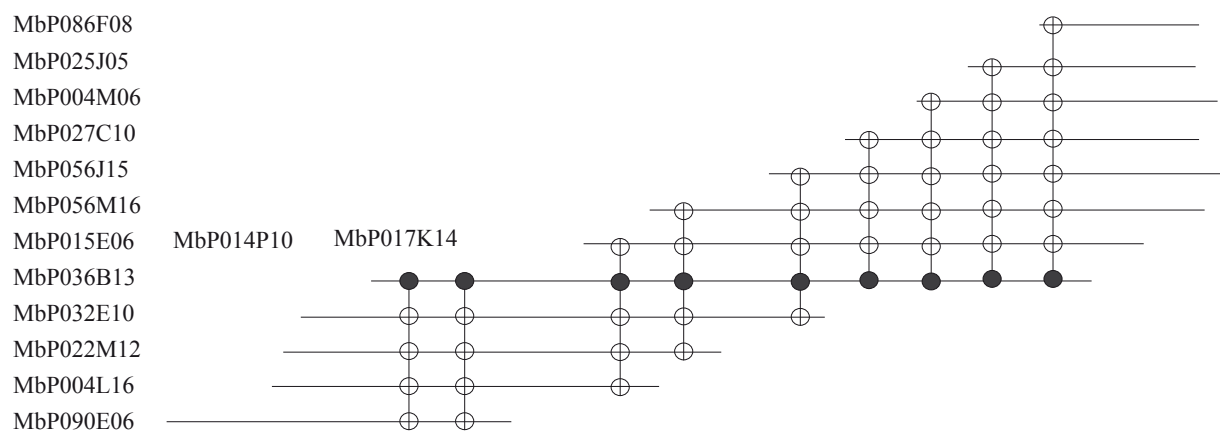

B

Supplement: Additional file 1 — MaRGA08 BAC fingerprints and contig assembly. A - EcoRI fingerprint of positive Musa babisiana BAC clones hybridized with the MaRGA08 probe. Lanes 1 to 22 correspond to BAC clones MbP004L16, MbP004M06, MbP012B09, MbP014P10, MbP015E06, MbP017K14, MbP019H11, MbP022M12, MbP025J05, MbP026I06, MbP027C10, MbP032E10, MbP032N20, MbP035J24, MbP036B13, MbP046G13, MbP053I03, MbP055C19, MbP056J15, MbP056M16, MbP086F08, MbP090E06, respectively. Lane M: 1 Kb ladder DNA marker. Sizes in Kb are indicated on the left. Black-white arrows indicate BAC contig groups. B - Resulting manually constructed contigs. BACs are represented by horizontal lines, circles represent hybridization bands in figure 1A. Bands of similar size are joined by vertical lines. BACs with all MaRGA08 hybridizations signals were chosen for sequencing (highlighted with black circles). [file 1471-2229-10-149-S1.PDF]

AAB Progeny IDN4x / PKW

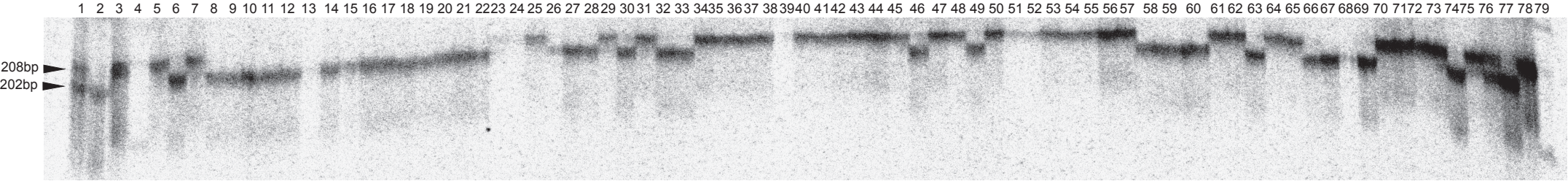

Supplement: Additional file 2 — Microsatellite mMaCIR341 segregation profile. The autoradiography of mMaCIR341 SSR amplification on the whole F1 mapping population from the cross PKW (BB) X IDN4x (AAAA) is presented. Controls are loaded on both sides of the gel: Parent PKW, lanes 1 and 76; BAC MbP036B13 (B2) lanes 2 and 77, BAC MbP026I06 (B1), lane 3 and 78; Parent IDN4x AAAA, lanes 4 and 79. Of the 71 individuals of the AAB F1 progeny, four have missing data (lanes 13, 24, 39, and 68), the remaining 67 exhibit one mMaCIR341 allele or the other demonstrating strict segregation of mMaCIR341 alleles at this locus. mMaCIR341 allele sizes are indicated on the left side. [file 1471-2229-10-149-S2.PDF]

A

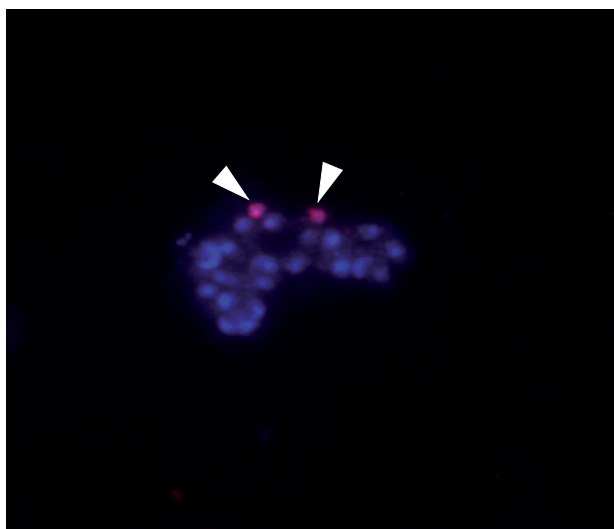

B

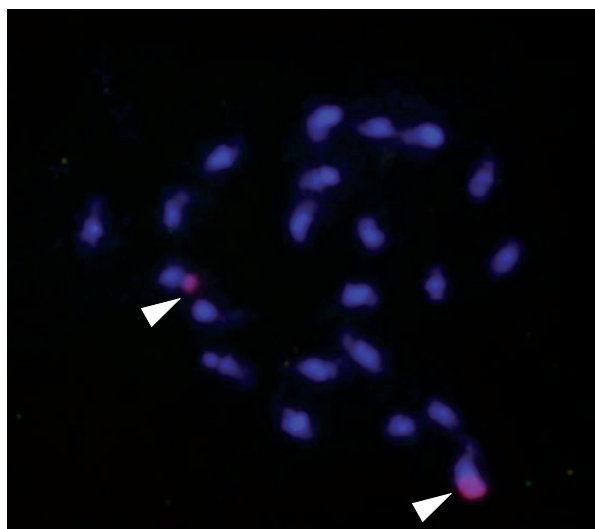

Supplement: Additional file 3 — Fluorescent in situ hybridization at the RGA08 locus of M. balbisiana. In situ FISH of metaphase spread chromosomes of M. balbisiana PKW using Biotin/Texas Red labelled BAC MbP036B13 (A) and 1.4 kb of MaRGA08 gene (B). Arrows indicate the two labelled chromosomes. [file 1471-2229-10-149-S3.PDF]

Coils output for B1\_g300 rga08K-1

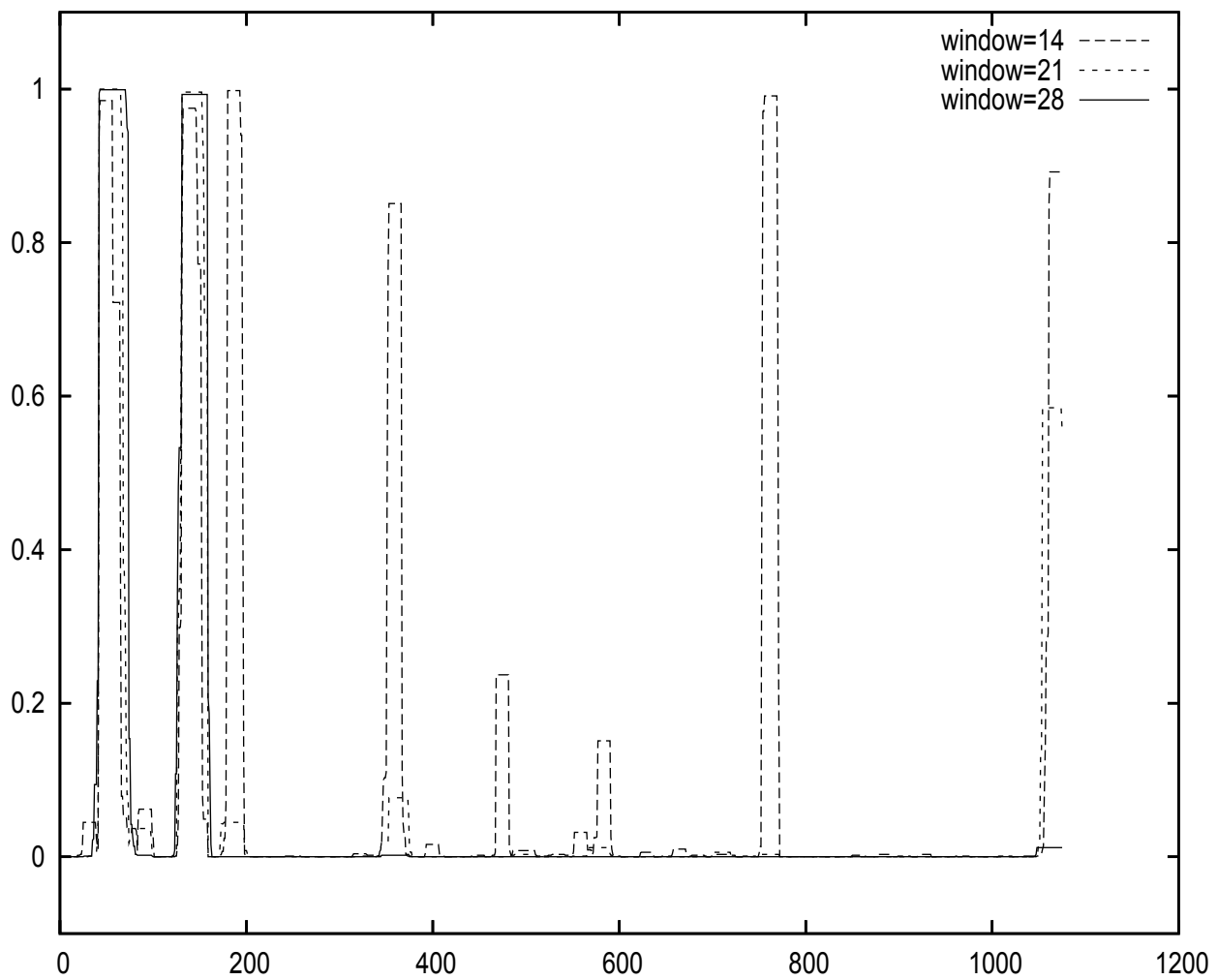

Supplement: Additional file 6 — Prediction of coiled-coil motifs in RGA08 predicted proteins. The probability of forming stable coiled-coils predicted by the COILS program http://www.ch.embnet.org/software/COILS_form.html is plotted against the amino acid residues of RGA08K-1. Predicted coiled-coil structures correspond to residues 34-54 and 123-143. [file 1471-2229-10-149-S6.PDF]
