# Supplementary material for: Mechanisms of haplotype divergence at the RGA08 nucleotide-binding leucine-rich repeat gene locus in wild banana (Musa balbisiana)
Source: BMC Plant Biol. 2010 Jul 16;10:149. doi: 10.1186/1471-2229-10-149 (PMC3017797; doi:10.1186/1471-2229-10-149)
Supplement: Additional file 4 — Gene list of MbP032N20c and MbP036B13 BAC sequences. The MbP032N20c and MbP036B13 polypeptides were aligned with BLASTALL to the rice proteome (MSU Annotation Release 6.0) to determine best hit relationships (BH). Rice genes used to infer syntenic relationships are in red. C, complete; Ψ pseudogene; f, fragment; N-f, N-terminal fragment;C-f, C-terminal fragment; r, remnant; N-r, N-terminal remnant; C-r, C-terminal remnant. [file 1471-2229-10-149-S4.PDF]

Locus_tag	C	Locus_tag	C	Product	Gene	BH *O. sativa*	E-value
MbP032N20cg010	N-f	-		Putative D-3-phosphoglycerate dehydrogenase precursor		LOC_Os06g44460	4.5E-58
MbP032N20cg020		MbP036B13g010	C-f	Putative aspartate carbamoyltransferase		LOC_Os08g15030	4.3E-154
MbP032N20cg025		MbP036B13g020		Histone H4	*H4*	LOC_Os01g61920	5.0E-56
MbP032N20cg030		MbP036B13g030		Conserved hypothetical protein		LOC_Os08g14620	2.1E-230
MbP032N20cg040	N-f	MbP036B13g040	N-f	Putative fasciclin-like arabinogalactan protein precursor		LOC_Os02g26320	5.5E-55
MbP032N20cg050		MbP036B13g050		Chlorophyll synthase	*VTE2*	**LOC_Os06g44840**	1.9E-145
MbP032N20cg070		MbP036B13g060		Hypothetical protein		No similarity found	
MbP032N20cg080		MbP036B13g070		Plasma membrane ATPase	*PMA*	LOC_Os04g56160	0
MbP032N20cg090		MbP036B13g080		Conserved hypothetical protein		LOC_Os04g56309	9.1E-14
MbP032N20cg100	N-r	MbP036B13g090	N-r	Villin N-terminal remnant		**LOC_Os06g44890**	2.9E-18
MbP032N20cg105		MbP036B13g100		Conserved hypothetical protein		**LOC_Os06g44900**	5.4E-168
MbP032N20cg110		MbP036B13g110		Conserved hypothetical protein		LOC_Os08g40500	1.1E-13
MbP032N20cg120		MbP036B13g120		Putative WRKY transcription factor		LOC_Os08g13840.2	7.5E-60
MbP032N20cg140		MbP036B13g140		Conserved hypothetical protein		LOC_Os02g07920	4.1E-52
MbP032N20cg150		MbP036B13g150		Conserved hypothetical protein		LOC_Os08g13350	1.4E-221
MbP032N20cg160		-		Disease resistance protein (CC-NBS-LRR)	*RGA08A*	LOC_Os11g45050	2.3E-163
MbP032N20cg165	r	-		RGA08 remnant	*RGA08B*	LOC_Os11g45180	2.1E-55
MbP032N20cg170	r	-		RGA08 remnant	*RGA08C*	LOC_Os11g45050	1.5E-69
MbP032N20cg180		-		Disease resistance protein (CC-NBS-LRR)	*RGA08D*	LOC_Os11g44960	3.0E-166
-		MbP036B13g160	ψ	Disease resistance protein (CC-NBS-LRR)	*RGA08S*	LOC_Os11g45050	2.0E-146
-		MbP036B13g165	C-r	MTERF C-terminal remnant	*MTERF5*	LOC_Os09g38720	2.8E-41
-		MbP036B13g170		Disease resistance protein (CC-NBS-LRR)	*RGA08T*	LOC_Os11g45180	3.4E-154
-		MbP036B13g180	N-r	MTERF N-terminal remnant	*MTERF6*	LOC_Os09g38720	1.2E-52
-		MbP036B13g190		Disease resistance protein (CC-NBS-LRR)	*RGA08U*	LOC_Os11g44960	1.1E-163
MbP032N20cg190	ψ	MbP036B13g260	ψ	MTERF domain-containing protein, mitochondrial	*MTERF1*	LOC_Os09g38720	2.9E-99
MbP032N20cg200	ψ	MbP036B13g270	ψ	Disease resistance protein (CC-NBS-LRR)	*RGA08E*	LOC_Os01g05620	1.4E-118
MbP032N20cg205	*r*	*MbP036B13g275*	*r*	*Gag-Pol remnant*			
MbP032N20cg210	ψ	-		Disease resistance protein (CC-NBS-LRR)	*RGA08F*	LOC_Os11g45090	7.5E-155
MbP032N20cg220	*N-f*	-		*Putative Gag-Pol polyprotein*			
MbP032N20cg225	*C-r*	*MbP036B13g195*	*C-r*	*Gag-Pol C-terminal remnant*			
MbP032N20cg240		MbP036B13g200		Disease resistance protein (CC-NBS-LRR)	*RGA08G*	LOC_Os11g45180	6.4E-169
MbP032N20cg250	ψ	MbP036B13g210	ψ	Disease resistance protein (CC-NBS-LRR)	*RGA08H*	LOC_Os11g45180	1.1E-139
MbP032N20cg260	C-r	MbP036B13g230	C-r	RPA1 C-terminal remnant	*RPA1*	LOC_Os06g40950	1.3E-68
MbP032N20cg280		MbP036B13g250		Disease resistance protein (CC-NBS-LRR)	*RGA08I*	LOC_Os11g45090	7.5E-163
MbP032N20cg285	*ψ*	-		*Putative Gag-Pol polyprotein*			
MbP032N20cg290		-		Disease resistance protein (CC-NBS-LRR)	*RGA08J*	LOC_Os11g45180	1.2E-164
-		MbP036B13g280	N-r	MTERF N-terminal remnant	*MTERF7*	LOC_Os09g38720	1.2E-47
-		MbP036B13g290	ψ	Disease resistance protein (CC-NBS-LRR)	*RGA08V*	LOC_Os11g45180	4.3E-165
MbP032N20cg300		MbP036B13g300		Disease resistance protein (CC-NBS-LRR)	*RGA08K*	LOC_Os11g45050	1.6E-167
MbP032N20cg310		-		Disease resistance protein (CC-NBS-LRR)	*RGA08L*	LOC_Os11g47780	2.2E-158
MbP032N20cg320		-		Disease resistance protein (CC-NBS-LRR)	*RGA08M*	LOC_Os11g45180	4.0E-163
MbP032N20cg330	ψ	-		MTERF domain-containing protein, mitochondrial	*MTERF2*	LOC_Os09g38720	1.8E-96
MbP032N20cg340	ψ	-		Disease resistance protein (CC-NBS-LRR)	*RGA08N*	LOC_Os11g13940	5.4E-141
MbP032N20cg360		-		Disease resistance protein (CC-NBS-LRR)	*RGA08O*	LOC_Os11g45180	1.0E-166
MbP032N20cg380		MbP036B13g310	ψ	Disease resistance protein (CC-NBS-LRR)	*RGA08P*	LOC_Os09g13820	3.4E-159
-		MbP036B13g315	C-r	MTERF C-terminal remnant	*MTERF8*	LOC_Os09g38720	1.8E-20
-		*MbP036B13g320*	*N-f*	*Putative Gag-Pol polyprotein*			
MbP032N20cg390	*f*	*MbP036B13g330*	*N-f*	*Putative Gag-Pol polyprotein*			
MbP032N20cg395	C-r	MbP036B13g325	C-r	MTERF C-terminal remnant	*MTERF3*	LOC_Os09g38720	9.5E-32
MbP032N20cg400	r	MbP036B13g340	r	RGA08 remnant	*RGA08Q*	LOC_Os11g45180	1.3E-60
MbP032N20cg410	N-r	-		RGA08 N-terminal remnant	*RGA08R*	LOC_Os11g45060	2.6E-108
-		MbP036B13g345	r	RGA08 remnant	*RGA08W*	LOC_Os09g13820	5.5E-77
-		MbP036B13g350		Disease resistance protein (CC-NBS-LRR)	*RGA08X*	LOC_Os11g45180	7.8E-167
MbP032N20cg420		MbP036B13g360		MTERF domain-containing protein, mitochondrial	*MTERF4*	LOC_Os09g38720	2.1E-145
MbP032N20cg440	N-f	MbP036B13g380		Putative serine/threonine-protein kinase		**LOC_Os06g45280**	5.5E-173
-		MbP036B13g390	N-f	Pseudouridylate synthase	*PUS*	**LOC_Os06g45250**	1.8E-63
