# Supplementary material for: Mechanisms of haplotype divergence at the RGA08 nucleotide-binding leucine-rich repeat gene locus in wild banana (Musa balbisiana)
Source: BMC Plant Biol. 2010 Jul 16;10:149. doi: 10.1186/1471-2229-10-149 (PMC3017797; doi:10.1186/1471-2229-10-149)
Supplement: Additional file 5 — Musa repetitive element (RE) similarity. Blastn hits were reported if the query coverage >50%. [file 1471-2229-10-149-S5.PDF]

Element	Length	Blastn hits		
		Acc. Num.	Description	Query coverage
RE1 Copia like	2882	AC186752.1	*Musa acuminata* clone MA4_64C22, complete sequence	54%
RE2 LARD « Clio »	4441	AP009334.1	*Musa balbisiana* clone BAC MBP_31O07, complete sequence	100%
		AC226045.1	*Musa acuminata* clone BAC MA4-71P20, complete sequence	100%
		AC226055.1	*Musa balbisiana* clone BAC MBP-8E4, complete sequence	100%
		AY484588.1	*Musa acuminata* clone MuG9, genomic sequence	100%
		AC186753.1	*Musa acuminata* clone MA4_54B05, complete sequence	100%
		AC226038.1	*Musa acuminata* clone BAC MA4-3F3, complete sequence	100%
		AC226035.1	*Musa acuminata* clone BAC MA4-1J14, complete sequence	100%
		AC226050.1	*Musa acuminata* clone BAC MAC-77E20, complete sequence	97%
		AC226047.1	*Musa acuminata* clone BAC MA4-86B3, complete sequence	97%
		AC226051.1	*Musa acuminata* clone BAC MAC-88K20, complete sequence	97%
		AC186751.1	*Musa acuminata* clone MA4_54N07, complete sequence	86%
RE3 Copia like « Mooz »	3821	AC186755.1	*Musa balbisiana* clone MBP_91N22, complete sequence	91%
		AC226035.1	*Musa acuminata* clone BAC MA4-1J14, complete sequence	55%
RE5 Copia like	4223	AC226035.1	*Musa acuminata* clone BAC MA4-1J14, complete sequence	63%
		AC226038.1	*Musa acuminata* clone BAC MA4-3F3, complete sequence	63%
		AC226041.1	*Musa acuminata* clone BAC MA4-4L11, complete sequence	53%
