# Supplementary material for: Mechanisms of haplotype divergence at the RGA08 nucleotide-binding leucine-rich repeat gene locus in wild banana (Musa balbisiana)
Source: BMC Plant Biol. 2010 Jul 16;10:149. doi: 10.1186/1471-2229-10-149 (PMC3017797; doi:10.1186/1471-2229-10-149)
Supplement: Additional file 7 — Real-time qPCR analysis of RGA08 expression. Quantitative Real-Time PCR was used to analyse the mRNA accumulation of: RGA08E, RGA08G, RGA08K, RGA08L, RGA08M (A) and RGA08O (B) in different tissues of PKW including peel of mature green fruit (Gpe), pulp of mature green fruit (Gpu), peel of ripe fruit (Rpe), pulp of ripe fruit (Rpu), flower (Fw), bract (Bc), root (Rt), seed (Se), stem (St), young leaf (Yl) old leaf (Ol). The y axis represents the relative fold difference of mRNA level and was calculated using the 2-ΔΔCt formula with actin as reference. The mRNA fold difference was relative to peel tissue of green fruit used as calibrator. Each data point is the mean of values obtained from qPCR reaction performed in triplicate on one sample. Each sample was prepared from tissues originated from two replicate plants or bunches. Vertical bars indicate standard deviation (S.D.). When no bar is shown, S.D. was smaller than the symbol. Primer pairs used in this study are indicated (C). [file 1471-2229-10-149-S7.PDF]

A

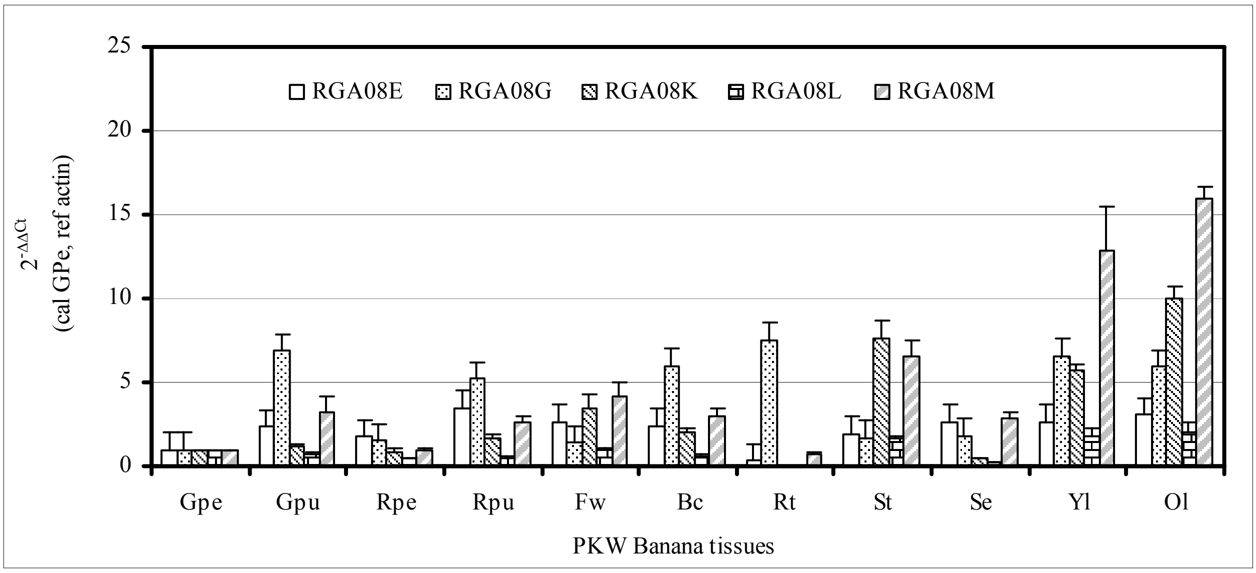

B

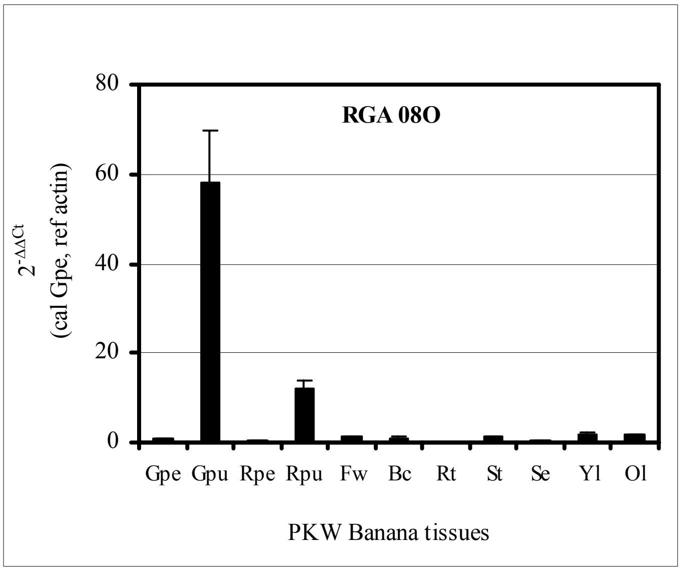

C

Targets	Names	Sequences	Product size (bp)
Actin	Act-F	5′- GAGAAGATACAGTGTCTGGA -3′	231
	Act-R	5′- ATTACCATCGAAATATTAAAAG -3′	
MbP32N20cg200	RGA08E-F	5′- ACGTCTTCTGGTTTTGGAGTGGCA -3′	194
	RGA08E-R	5'- CGCAACCAACCTCGATATCTGTGC -3'	
MbP32N20cg240	RGA08G-F	5′- TCGGCAGCAGGATAGCAGTAGCA -3′	167
	RGA08G-R	5′- GATGCCGCCAAAATGGCCCG -3′	
MbP32N20cg300	RGA08K-F	5′- AGATGCAGACCTGTGAGTCTGTCAA -3′	248
	RGA08K-R	5'- TGAGTGTTTTGCAGTATGCACGTTTC -3'	
MbP32N20cg310	RGA08L-F	5′- ACGTCTTCTGGTTTTGGAGTGGCA -3′	193
	RGA08L-R	5'- GCAGCCAACCTATCGATCTGCG -3'	
MbP32N20cg320	RGA08M-F	5′- CATGCTCACGTCTCGACCATC -3′	151
	RGA08M-R	5'- TGGTAAATATCATGGAAGCCGTGAA -3'	
MbP32N20cg360	RGA08O-F	5′- ACGTCTTCTGGTTTTGGAGTGGCA -3′	194
	RGA08O-R	5′- CGCAACCAACCTCTCGATCTGCAT -3′	
