# Supplementary material for: Mechanisms of haplotype divergence at the RGA08 nucleotide-binding leucine-rich repeat gene locus in wild banana (Musa balbisiana)
Source: BMC Plant Biol. 2010 Jul 16;10:149. doi: 10.1186/1471-2229-10-149 (PMC3017797; doi:10.1186/1471-2229-10-149)
Supplement: Additional file 8 — Estimation of divergence time between PKW haplotypes. Gene names are those listed in Additional File 4, sequence length is given in base pairs, Sd is the number of synonymous mutations, p-distance is estimated as the ratio of synonymous mutations per synonymous sites [41] and divergence time is calculated using a synonymous substitution rate of 0.45 per 109 year [15]. [file 1471-2229-10-149-S8.PDF]

Gene	Length	Sd	p-distance	Divergence
Aspartate carbamoyltransferase	519	3	0.0240	2.67
Conserved hypothetical protein (MbP032N20cg030)	655	0	0.0000	0.00
Chlorophyll synthase	1185	3	0.0103	1.14
Hypothetical protein	339	0	0.0000	0.00
Plasma membrane ATPase	2865	7	0.0106	1.18
Conserved hypothetical protein (MbP032N20cg090)	324	0	0.0000	0.00
Conserved hypothetical protein (MbP032N20cg105)	1440	1	0.0029	0.32
Conserved hypothetical protein (MbP032N20cg110)	315	2	0.0253	2.81
WRKY transcription factor	732	1	0.0055	0.61
Conserved hypothetical protein (MbP032N20cg150)	1461	9	0.0246	2.73
Mitochondrial transcription termination factor	1833	1	0.0024	0.27
Serine threonine protein kinase	1211	0	0.0000	0.00
Mean	12879	27	0.0088	0.98
